# The transcription factor CsS40 negatively regulates *TCS1* expression and caffeine biosynthesis in connection to leaf senescence in *Camellia sinensis*

**DOI:** 10.1093/hr/uhad162

**Published:** 2023-08-10

**Authors:** Xinzhuan Yao, Hufang Chen, Antao Ai, Fen Wang, Shanshan Lian, Hu Tang, Yihe Jiang, Yujie Jiao, Yumei He, Tong Li, Litang Lu

**Affiliations:** College of Tea Sciences, Institute of Plant Health & Medicine, The Key Laboratory of Plant Resources Conservation and Germplasm Innovation in Mountainous Region (Ministry of Education), Guizhou University, Guiyang 550025, China; College of Tea Sciences, Institute of Plant Health & Medicine, The Key Laboratory of Plant Resources Conservation and Germplasm Innovation in Mountainous Region (Ministry of Education), Guizhou University, Guiyang 550025, China; College of Tea Sciences, Institute of Plant Health & Medicine, The Key Laboratory of Plant Resources Conservation and Germplasm Innovation in Mountainous Region (Ministry of Education), Guizhou University, Guiyang 550025, China; School of Biological Science and Agriculture, Qiannan Normal University for Nationalities, Duyun 558000, China; College of Tea Sciences, Institute of Plant Health & Medicine, The Key Laboratory of Plant Resources Conservation and Germplasm Innovation in Mountainous Region (Ministry of Education), Guizhou University, Guiyang 550025, China; College of Tea Sciences, Institute of Plant Health & Medicine, The Key Laboratory of Plant Resources Conservation and Germplasm Innovation in Mountainous Region (Ministry of Education), Guizhou University, Guiyang 550025, China; College of Tea Sciences, Institute of Plant Health & Medicine, The Key Laboratory of Plant Resources Conservation and Germplasm Innovation in Mountainous Region (Ministry of Education), Guizhou University, Guiyang 550025, China; College of Tea Sciences, Institute of Plant Health & Medicine, The Key Laboratory of Plant Resources Conservation and Germplasm Innovation in Mountainous Region (Ministry of Education), Guizhou University, Guiyang 550025, China; College of Tea Sciences, Institute of Plant Health & Medicine, The Key Laboratory of Plant Resources Conservation and Germplasm Innovation in Mountainous Region (Ministry of Education), Guizhou University, Guiyang 550025, China; College of Tea Sciences, Institute of Plant Health & Medicine, The Key Laboratory of Plant Resources Conservation and Germplasm Innovation in Mountainous Region (Ministry of Education), Guizhou University, Guiyang 550025, China; College of Tea Sciences, Institute of Plant Health & Medicine, The Key Laboratory of Plant Resources Conservation and Germplasm Innovation in Mountainous Region (Ministry of Education), Guizhou University, Guiyang 550025, China

## Abstract

Caffeine is considered as one of the most important bioactive components in the popular plant beverages tea, cacao, and coffee, but as a wide-spread plant secondary metabolite its biosynthetic regulation at transcription level remains largely unclear. Here, we report a novel transcription factor *Camellia sinensis Senescnece 40* (*CsS40*) as a caffeine biosynthesis regulator, which was discovered during screening a yeast expression library constructed from tea leaf cDNAs for activation of tea caffeine synthase (*TCS1*) promoter. Besides multiple hits of the non-self-activation CsS40 clones that bound to and activated *TCS1* promoter in yeast-one-hybrid assays, a split-luciferase complementation assay demonstrated that *CsS40* acts as a transcription factor to activate the *CsTCS1* gene and EMSA assay also demonstrated that CsS40 bound to the *TCS1* gene promoter. Consistently, immunofluorescence data indicated that CsS40-GFP fusion was localized in the nuclei of tobacco epidermal cells. The expression pattern of *CsS40* in ‘Fuding Dabai’ developing leaves was opposite to that of *TCS1*; and knockdown and overexpression of *CsS40* in tea leaf calli significantly increased and decreased *TCS1* expression levels, respectively. The expression levels of *CsS40* were also negatively correlated to caffeine accumulation in developing leaves and transgenic calli of ‘Fuding Dabai’. Furthermore, overexpression of *CsS40* reduced the accumulation of xanthine and hypoxanthine in tobacco plants, meanwhile, increased their susceptibility to aging. *CsS40* expression in tea leaves was also induced by senescence-promoting hormones and environmental factors. Taken together, we showed that a novel senescence-related factor CsS40 negatively regulates *TCS1* and represses caffeine accumulation in tea cultivar ‘Fuding Dabai’. The study provides new insights into caffeine biosynthesis regulation by a plant-specific senescence regulator in tea plants in connection to leaf senescence and hormone signaling.

## Introduction

Alkaloids are one of the most significant secondary metabolites in plants and display a plethora of biological activities in medicines and defensive roles in plants against herbivores and pathogens [1]. Alkaloids, such as vincristine, nicotine, and caffeine, have been used for thousands of years as medicine, dietary, and recreational neural stimulants. As the most widely consumed bioactive alkaloid of plant origin [2], caffeine accounts for 2–5% and 1–2.7% of the dry weight of tea and coffee, respectively [3] as the core supplementary to many non-alcoholic beverages. As an effective neurostimulant, caffeine can boast a plethora of pharmacological effects, including the promotion of diuresis and digestion and a reduction in hypertension and anxiety [4]. Caffeine accumulates in many plants and exerts a protective role against attacks by fungal and bacterial pathogens or herbivores, and serves as a signaling molecule for communication with neighboring plants [5–7]. Thus caffeine has attracted increasing attention on metabolic engineering and biosynthesis mechanism in plants.

Caffeine is synthesized in young plant tissues, but predominantly in flowers, young leaves, and seeds [8–10]. At least three possible biosynthetic pathways have been identified for caffeine biosynthesis in plants [11–13]; however, the adenine content in tea plant cells is much higher than that of guanine, indicating that the major route for caffeine biosynthesis is from adenine [12, 13]. In the common caffeine-specific biosynthesis pathway from xanthosine → 7-methylxanthosine → 7-methylxanthine → theobromine → caffeine [14], various N-methyltransferases (*NMT*) expanded in tea genome facilitate the transfer of methyl groups to N-7, N-3, and N-1 positions of xanthine backbones and are critically involved in caffeine biosynthesis [15]. Among them, tea caffeine synthase 1(*TCS1*) is a key enzyme in tea plants that catalyzes both 7-methylxanthine N-3 methyltransferase (theobromine synthase, TS, EC 2.1.1.159) and theobromine N-1 methyltransferase (caffeine synthase, CS, EC 2.1.1.160) reactions [2]. *TCS1* gene silencing in transformed somatic embryos drastically reduced both theobromine and caffeine contents [16]. Additionally, data regarding genetic variations in the *TCS1* gene in tea plant populations support a key role of *TCS1* in determining caffeine content [17]. Moreover, the low caffeine content in cross-grafted *Camellia sinensis* and *Camellia oleifera* tea plants was attributed to low expression levels of *TCS1* and the low accumulation of CS [18]. These results indicate that *TCS1* is crucial for caffeine biosynthesis and the manipulation of *TCS1* may help create new tea plant variants with differing caffeine contents. As a highly bioactive natural product, caffeine biosynthesis in plants is tightly regulated at transcription and posttranscriptional levels in a spatiotemporal manner [3, 15, 17, 19, 20]. Various transcription factors (TFs), including *WRKY*, *MYB*, *MYC*, *bZIP*, *NAC*, *bHLH*, *DUF*, and *AP2/ERF*, had been proposed to target the *TCS1* gene and regulate *TCS1* expression [3, 15], because caffeine accumulation in tea plants are subject to regulations by various phytohormones and environmental cues. However, only one NAC and a MYB factor were characterized to regulate *TCS1* expression in tea plants [15, 17]. Therefore, more TFs need to be identified and characterized to understand the complex regulation of caffeine biosynthesis in tea plants. Here, by screening a yeast protein expression library from tea leaf for activator of *TCS1* (TEA015791, the major caffeine-determination loci) promoter [9, 21, 22], and conducting various biochemical assays and genetic examination of loss-of-function and gain-of-function mutants in tea tissues and tobacco plants, we screened out and characterized a senescence-regulation related unknown TF S40 protein as a novel *TCS1* expression and caffeine biosynthesis regulator in tea plant. Overexpression and silencing of *CsS40* in genetically transformed tobacco plants and tea leaf calli showed correspondingly altered contents of purine alkaloids in these materials, further proving the regulatory function of *CsS40* in *TCS1* expression and caffeine biosynthesis, and in the promotion of leaf senescence in tobacco plants. and the study provides new insight into the novel regulatory mechanism of caffeine biosynthesis in tea plants.

## Results

### Expression pattern of *TCS1* and pro-*TCS1* in tea plant tissue

**Figure 1:**
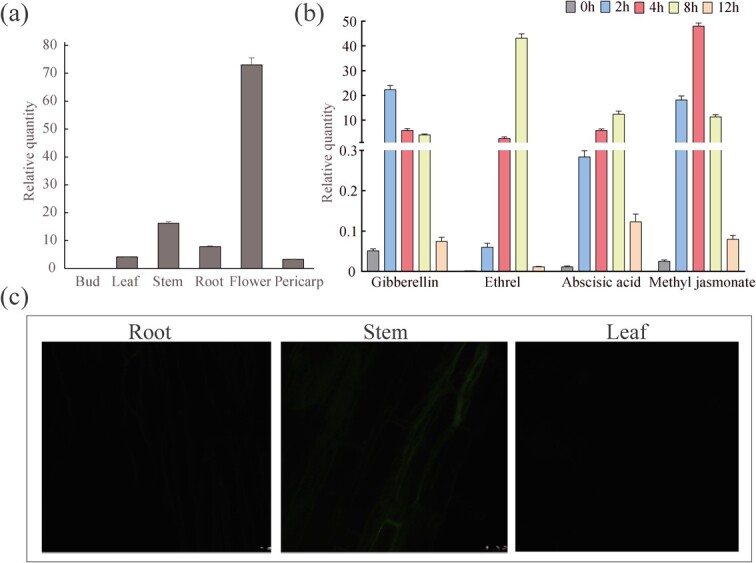
Expression patterns of *TCS1* in ‘Fuding Dabaicha’s. (**a**) Model of shoot sampling, which included the flower, stem, root, leaf, pericarp, and bud. (**b**) The relative expression level of *TCS1* in tea leaves sprayed with a solution containing gibberellin, ethrel, abscisic acid, and methyl jasmonate. (**c**) The fluorescence distribution of the Pro-TCS1-GFP protein in the roots, stems, and leaves of transgenic tobacco plants as observed by confocal fluorescence microscopy.

The *TCS1* promotor (pro-*TCS1*) sequence contained many functional *cis*-elements (Fig. 3d). To test how the expression patterns of *TCS1* in tea plant tissues were affected by hormone-mediated light regulation and other factors, RT-qPCR was conducted and results demonstrated that the relative higher expression level of *TCS1* was observed in flower > stem > root > leaf > pericarp > bud (Fig. 1a), and that *TCS1* transcripts in tea leaves were up-regulated rapidly and then decreased to basal levels after being sprayed with solutions containing 1 mol/L gibberellin, 0.1 mmol/L ethrel, 0.1 mmol/L abscisic acid, and 0.1 mmol/L methyl jasmonate, respectively, as compared with control leaves sprayed with water only (Fig. 1b).

The complete *TCS1* gene, including *pro-TCS1* and ORF, was inserted into the pCAMBIA1300-T-GFP vector driven by a 35S promoter to generate the recombinant 35S-*pro*-*TCS1*::*GFP* plasmid, which was used to transform *A. tumefaciens* LBA4404. Tobacco plants were subsequently transformed with the positive Agrobacteria transformant. The resulting transgenic tobacco plants were examined under a confocal fluorescence microscope. The GFP signal intensity was the highest in the stem, and low in the root and leaf, which are consistent with the relative expression of *TCS1* in tea plant tissues (Fig. 1c).

### Discovery of a candidate transcription factor for regulating *TCS1*

**Figure 2:**
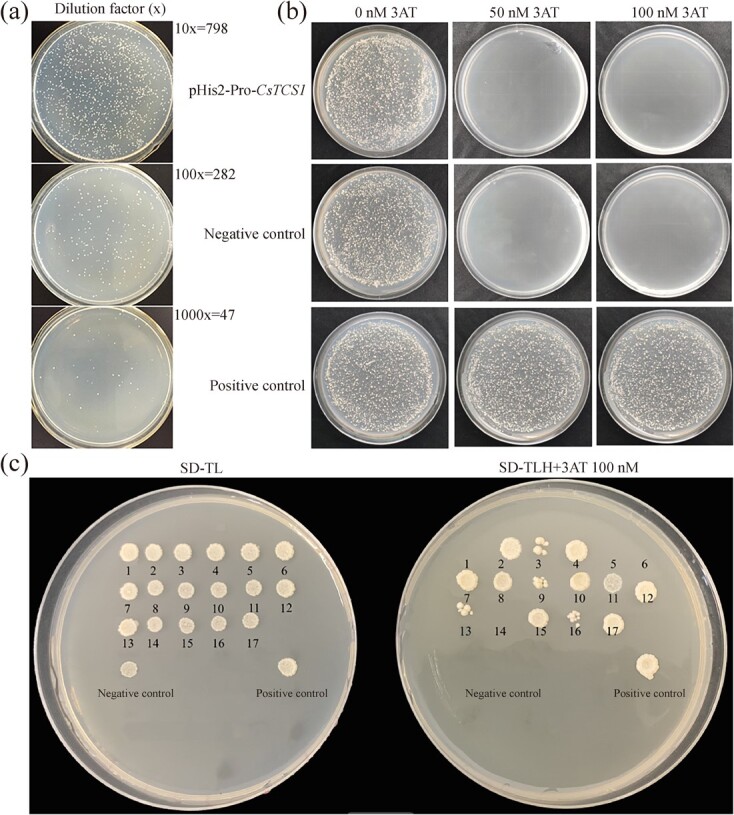
Construction of a cDNA library and screening of transcription factors using Y1H. (**a**) Screening efficiency of the *CsTCS1* promotor library; the dilution factor from top to bottom is 10×, 100×, and 1000×, respectively. (**b**) Results of self-activation detection of bait colonies, 3-amino-1,2,4-triazole (3-AT) resistant yeast bait strains grown on SD/−Trp-Leu-His (SD-TLH) media supplemented with different concentrations of 3-AT (0, 50, 100 nM). (**c**) Screening of the constructed cDNA library for transcription factors on SD-TLH media containing 50 nM 3-AT. Negative controls, pGAD53m + pHis2; positive controls, pGAD53m + p53His.

To elucidate transcription factors that control the expression of *TCS1* in tea plants, a tea leaf protein expression library was constructed in a yeast expression library system pGADT7 for use in a yeast-one hybrid assay to screen for *TCS1* promoter activators. Success of the tea leaf yeast expression library was verified by the calculated capacity of 4.44 × 10^4^ CFU (Colony-Forming Units- (Fig. 2a). To determine whether the *TCS1* promoter has self-activation activity, transcriptional activator assays were performed in yeast Y187 cells with the 1570 bp of *TCS1* promotor fragment from tea cultivar ‘Fudingdabai’ genome cloned into the pHis2 vector as the *pro-TCS1* bait construct. The yeast Y187 cells transformed with the bait plasmid were able to grow on SD-TL media lacking 3-AT, and so did positive and negative controls. However, while the positive control was able to grow on SD-TLH media containing 3-AT, neither pHis2-*pro*-*TCS1* nor the negative control were able to grow in the presence of 3-AT, indicating that the pHis2-*pro*-*TCS1* did not display self-activation activity (Fig. 2b). Moreover, the Y187 yeast strain harboring the bait plasmid was unable to grow on SD-TLH medium containing ≥50 mM 3-AT (Fig. 2b); thus, 50 mM 3-AT was added to the SD-TLH medium for the screening of the tea leaf yeast expression library for the potential transcription factors.

For screening, the replica plating of the yeast library was carried out using flanged cloth on day 3 of activation cultivation, and the plates were incubated for 7–14 days. A total of 188 positive transformants on SD-TLH media containing 50 mM 3-AT were selected and transferred to SD-TL medium for continued cultivation for 2–3 days. Seventeen initially well-grown positive colonies were obtained, which were transferred, along with negative and positive controls, to SD-TL medium in the presence of 50 mM 3-AT for further incubation at 30°C for 3–4 days. The initially positive colonies survived on the SD-TL media in the presence of 50 mM 3-AT for plasmid extraction and sequencing analysis in transformed *Escherichia coli* TOP10 competent cells (Fig. 2c). The negative control did not activate the His reporter gene and therefore did not survive on either media. Nine positive clones in total did activate the His reporter gene and survived on both media.

### Evaluation of *CsS40* obtained from the cDNA library

These positive clones were sequenced and BLAST against the NCBI and GenBank database. These nine positive clones belonged to genes encoding nine different proteins (Table S1, see online supplementary material), of which XP_028075486.1 (No. 17) was a putative candidate transcription factor for the *TCS1* promoter in tea plants. XP_028075486.1 had 1158 bp in length, encoding a polypeptide of 212 amino acid residues with the molecular weight of 23.37 kD and theoretical isoelectric point as 5.98. It possessed a conserved structural domain of the Senescence_reg S40 gene superfamily, annotated as *C. sinensis* uncharacterized LOC114277741 (CSS0010485.1). To the best of our knowledge, there exist no related studies in tea plants on the protein; we thus named this gene as *CsS40*. The promoter sequence of the *CsS40* contains many *cis*- elements such as ARE, Circadian, ABRE, and GARE-motif (Fig. S1, see online supplementary material).

### *CsS40* transcription activated the *TCS1* promoter

**Figure 3:**
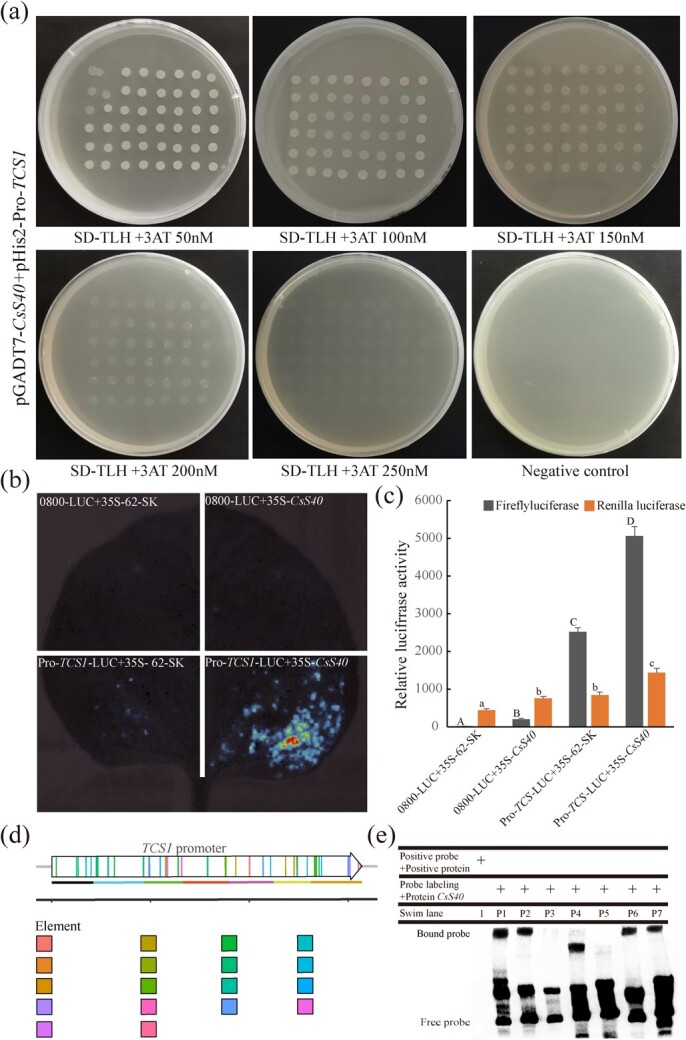
Validation of the transcriptional activity of *CsS40*. (**a**) Detection of the interaction between *CsS40* and the *TCS1* promoter. Yeast cells expressing the pGADT7-BD vector were used as a negative control. (**b**) Pro-*TCS1* was shown to associate with *CsS40* using a split-luciferase complementation assay. *Nicotiana benthamiana* leaves were transformed with *Agrobacterium* EHA105 cells harboring the Pro-*TCS1*-nLUC and cLUC-35S:*CsS40* plasmids. The combination of Pro-*TCS1*-nLUC and cLUC-35S: *CsS40* resulted in robust luciferase complementation; however, the negative control produced no obvious signal. (**c**) In a split luciferase complementation assay, *N. benthamiana* leaves were transformed by injection of Agrobacterium EHA105 cells harboring Pro-*TCS1*-nLUC and cLUC-35S:*CsS40* plasmids. the letters A-d and A-c indicate whether there is significant difference (*P* < 0.05). (**d**) Different DNA fragments and *cis*-acting elements of promoter. (**e**) EMSA verified the interaction between CsS40 and promoter of *TCS1.*1: positive probe+ positive protein; P1–P7*:* binding of different DNA fragments of promoters to S40 protein.

To further verify the activation function of CsS40, the CsS40 coding sequence from the recombinant pGADT7-*CsS40* plasmid was re-constructed and inserted into the pGADT7 vector for re-transformation of Y187 yeast strain together with pHis2-pro-*TCS1*. The positive clones were grown on SD-TLH medium supplemented with 3-AT (50, 100, 150, 200, and 250 mM) [23] (Fig. 3a) for 3–4 days, and most yeast clones grew well, indicating that CsS40 can bind and activate pro*-TCS1*.

We then used a split-luciferase complementation assay. The pPro-*TCS1* was fused to the N-terminus of luciferase to generate Pro-*TCS1*-nLUC, and *CsS40* was fused to the C-terminus of luciferase to generate cLUC-*CsS40*, both of which were used to transform *N. benthamiana* leaves. Luciferase activity was observed following co-expression of Pro-*TCS1*-nLUC and CLU-*CsS40*, while no luciferase activity was observed following expression of the negative control pGreenII 62-SK (Fig. 3b and c). These results indicate that *CsS40* has a regulatory effect on the activation of the *TCS1* promoter. DNA-protein interaction can be verified with electrophoretic mobility shift assay (EMSA) to further examine the interaction between *TCS1* promoter and CsS40 protein. Incubate the purified CsS40 protein with different DNA fragments of biotin labeled *TCS1* promoter (Fig. 3d) before subjecting it to gel migration experiments. In Fig. 3e, it can be seen that after binding of CsS40 protein with different lengths of nucleic acid fragments, the swim lane strip of protein + nucleic acid significantly lagged behind the swim lane with only probes. In swim lane P1-P7, the gel electrophoresis results of probes with different lengths interacted with proteins were varying differently (P1–P7), but it clearly showed that the CsS40 protein can bind to (P1,P2,P4,P6,P7) the *TCS1* promoter segment, and P4 may be too small, leading to a too-fast migration speed. The absence of P3 and P5 binding to S40 protein may be due to the absence of obvious binding sites.

### Subcellular localization of CsS40

**Figure 4:**
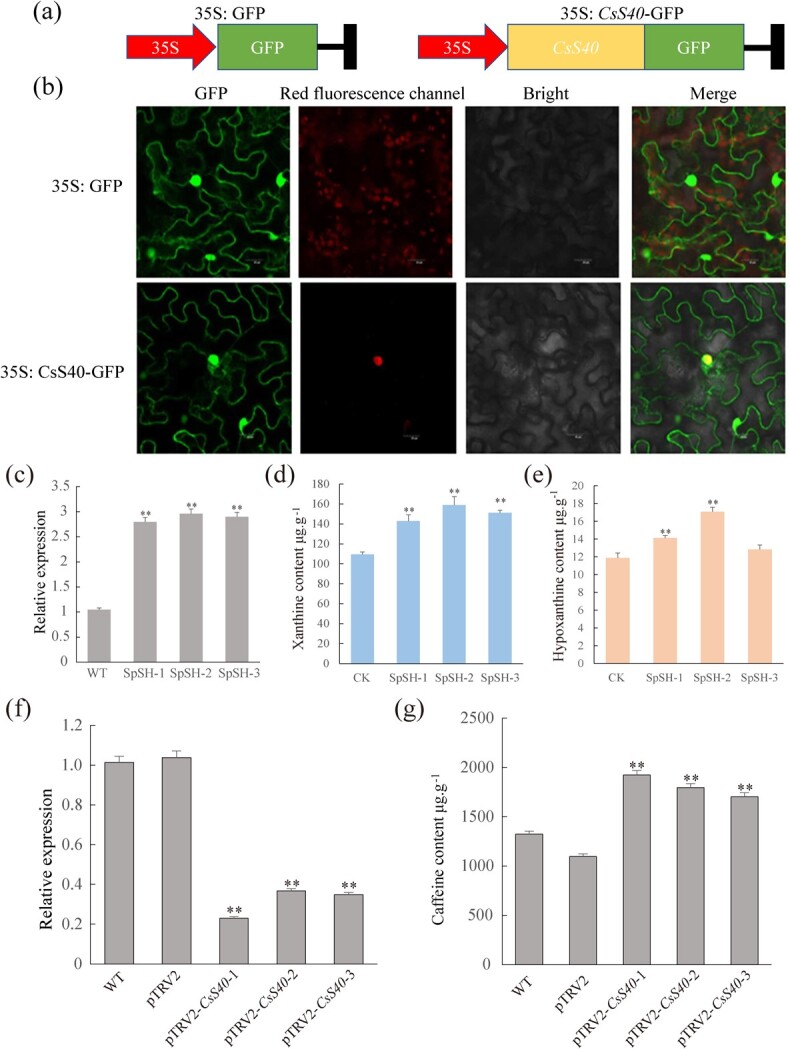
Subcellular localization of *CsS40* gene and the effect of overexpression of *CsS40* on the accumulation of caffeine precursors xanthine and hypoxanthine in tobacco*.* (**a**) Schematic of the vectors used to assess *CsS40* subcellular localization. (**b**) Detection of GFP fluorescence intensity. 35S: GFP, empty vector: GFP expression driven by the 35S promoter; 35S: *CsS40*-GFP, *CsS40*-GFP fusion protein driven by the 35S promoter. (**c**) RT-qPCR was used to evaluate the expression levels of *CsS40* in tobacco. CK, transgenic tobacco overexpressing pSH737-35S-GUS as a control; NtpSH, transgenic tobacco overexpressing pSH737-35S-*CsS40-*GUS. (**d**) HPLC was used to evaluate the xanthine content of NtpSH tobacco. (**e**) HPLC was used to evaluate the hypoxanthine content of NtpSH tobacco (*P < 0.05; **P < 0.01). (**f**) *CsS40* was silenced using VIGS; RT-qPCR was performed to evaluate the relative expression of *CsS40* in transformed seedlings. (**g**) *CsS40* was silenced using VIGS; HPLC was performed to evaluate the accumulation of caffeine in transformed seedlings (*P < 0.05; **P < 0.01).

Transient expression of *CsS40* in tobacco leaf epidermal cells was conducted to determine its subcellular localization. The ORF of *CsS40* was subcloned into the GFP fusion vector pCAMBIA1300-*35S-GFP* (35S: GFP) (Fig. S2, see online supplementary material) to generate the *35S* promoter-driven recombinant CsS40-GFP fusion expression vector (Fig. 4a). The sequencing-confirmed construct was transformed into *A. tumefaciens* GV3101 for subsequent infiltration into tobacco leaves. The GFP signal intensity of CsS40-GFP was clearly distributed in the nucleus and cell membrane, while that of the empty vector was diffusely located in the cell membrane, cytoplasm, and nucleoplasm (Fig. 4b).

**Figure 5:**
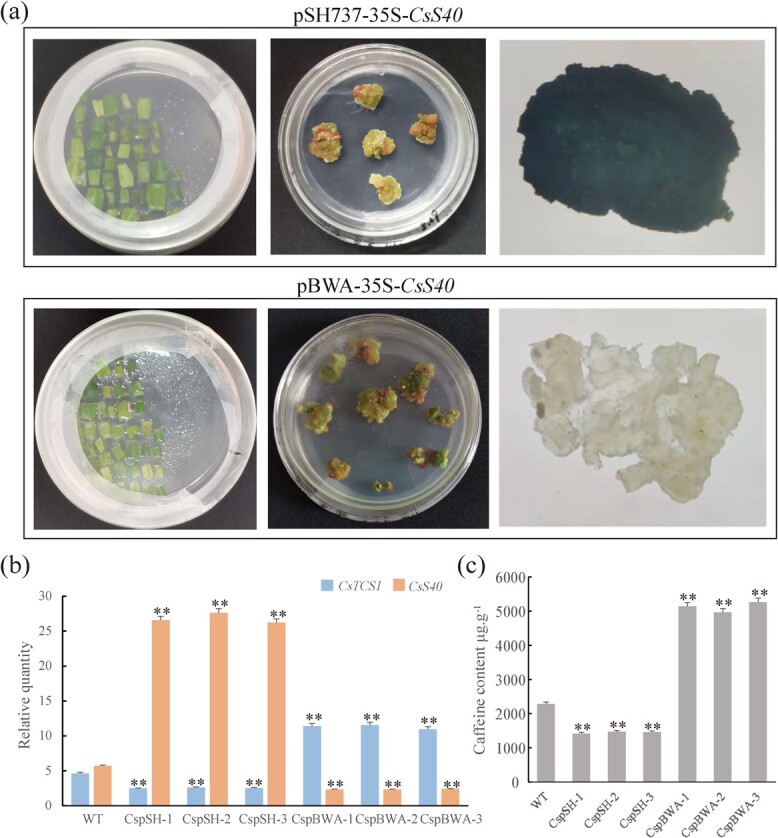
Effect of the overexpression and silencing of *CsS40* in tea calli on the expression of *TCS1* and the accumulation of caffeine. (**a**) Calli transformed with the pSH737-35S-*CsS40* overexpression plasmid or the pBWA-35S-*CsS40* silencing plasmid were cultured on MS medium containing 0.5 mg/L 6-BA, 1.0 mg/L NAA, 200 mg/L Tim, and 30 mg/L Kan. Subsequently, GUS staining of transgenic tea plant calli was performed. (**b**) RT-qPCR was used to evaluate the expression levels of *CsS40* and *TCS1* in calli. WT, non-transgenic callus as a control; CspSH, transgenic callus overexpressing *CsS40*; CspBWA, transgenic callus harboring silenced *CsS40*; **, extremely significant differences between values. (**c**) HPLC analysis of caffeine content in callus of CspSH and CspBWA.

### Overexpression of *CsS40* in tobacco plants reduced the accumulation of xanthine and hypoxanthine

To understand the biological function of CsS40, tobacco leaf disks were infected with *A. tumefaciens* harboring the *pSH737-35S*-*CsS40-*GUS, and *pSH737-35S-GUS* (as an empty vector control) overexpression plasmids individually, and the resistant positive plants were selected by kanamycin resistance marker (Fig. S3, see online supplementary material; Fig. 4c). The transgenic tobacco lines overexpressing *pSH737-35S*-*CsS40*-GUS vector were named as NtpSH and the tobacco lines overexpressing *pSH737-35S-GUS* vector were named as control (CK). The expression level of *CsS40* in NtpSH was 2.66-times higher than that in CK tobacco plants, indicating the successful overexpression of *CsS40.* Moreover, the accumulation of xanthine and hypoxanthine was determined by HPLC, despite no caffeine detected in transgenic tobacco leaves. The average contents of xanthine in NtpSH plants were 151.01 μg/g, which was 1.38 times higher than that in CK plants (CK = 109.40 μg/g) (Fig. 4d). The average contents of hypoxanthine in NtpSH plants were 14.67 μg/g, which was 1.23 times higher than that in CK (CK = 11.88 μg/g) (Fig. 4e). Thus, *CsS40* overexpression increased the accumulation of caffeine precursors xanthine and hypoxanthine in transgenic tobacco plants.

### Silencing of *CsS40* repressed the formation of caffeine in ‘Fuding Dabaicha’

To further understand the function of CsS40 in tea plants, we repressed the *CsS40* transcription factor gene in ‘Fuding Dabaicha’ leaf tissues by using VIGS technology, which was established previously [24]. The relative expression of *CsS40* was assessed by RT-qPCR and the caffeine content was determined by using HPLC. In comparison with the wild-type plants and those expressing empty vector, the relative expression of *CsS40* in tea plant leaves expressing PTRV2-*CsS40* was significantly decreased to 36.19% of the wild-type and empty vector control, the caffeine content was correspondingly 1.29 times higher (Fig. 4f and g). These data demonstrated that silencing of *CsS40* also repressed the formation of caffeine in ‘Fuding Dabaicha’, thus CsS40 is a negative regulator in caffeine biosynthesis in tea plants.

### Overexpression and silencing of *CsS40* in tea calli affected the accumulation of caffeine

To further verify the function of CsS40 in regulating the expression of *CsTCS1* and the accumulation of caffeine in tea plants, the ORF of *CsS40* was cloned into the *pSH737-35S* overexpression vector and the pBWA(V)K-cas9pl(atu6i)-35S silencing vector. *A. tumefaciens* strain LBA4404 was transformed with these plasmids individually using the freeze–thaw method. Tea leaf calli were infected with *A. tumefaciens* harboring *pSH737-35S-CsS40* and *pBWA(V)K-cas9pl(atU6i)-CsS40* vectors, respectively, for the overexpression and silencing of *CsS40*, and the positive transformants were selected on kanamycin-containing media (Fig. 5a). Successful transformation was also verified by PCR of genomic DNA from positive calli. The tea calli overexpressing *CsS40* were designated as CspSH, while the tea calli with silenced *CsS40* were named as CspBWA (Figs S4 and S5, see online supplementary material).

Total RNAs were isolated from CspSH and CspBWA calli, and the relative expression of *CsTCS1* and *CsS40* were examined with qRT-PCR (Fig. 5b). The expression levels of *CsS40* in CspSH calli were averagely 4.71 times higher than that in wild-type calli, while the expression level of *TCS1* in CspSH calli was 54.39% of that in wild-type calli, indicating that overexpression of *CsS40* in tea plant leaf calli repressed the expression of *TCS1*. Moreover, the expression levels of *TCS1* and *CsS40* in CspBWA calli were 2.46 times higher than and 41.22% of that in wild-type calli, respectively, indicating that the silencing of *CsS40* in tea plant leaf calli increased the expression level of *CsTCS1*. Thus, CsS40 negatively regulated the *TCS1* expression of *in* tea leaf callus. Subsequently, the accumulation of caffeine in tea leaf calli was evaluated (Fig. S6, see online supplementary material). The average caffeine content of CspSH calli was 1453 μg/g, a decrease by only 36.41% of the wild-type (WT: 2285 μg·g^−1^). However, the average caffeine content in CspBWA calli increased to 5123 μg/g, which was 1.24-fold higher than that in the wild-type calli (Fig. 5c). Thus, overexpression of *CsS40* inhibited the *TCS1* expression and thereafter the accumulation of caffeine in tea leaf calli, while silencing of *CsS40* promoted the *TCS1* expression and thereafter increased the accumulation of caffeine. In summary, *CsS40* negatively regulates *TCS1* and inhibits caffeine accumulation in ‘Fuding Dabaicha’.

### Overexpression of *CsS40* rendered tobacco plants more susceptible to senescence

**Figure 6:**
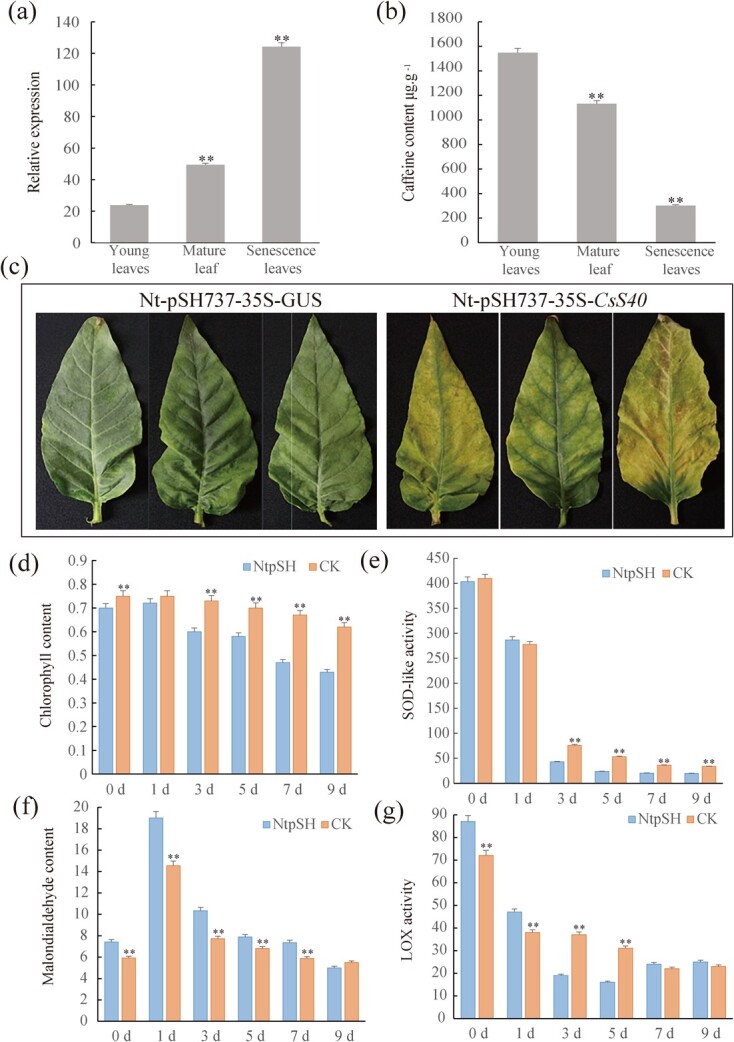
Overexpression of *CsS40* affects senescence resistance in tea plants. (**a**) Model of shoot sampling, including the young leaves, the mature leaves, and the senescent leaves. (**b**) The relative expression of *CsS40* in the leaves of ‘Fuding Dabaicha’s with differing degrees of senescence. (**c**) Caffeine content in the leaves of ‘Fuding Dabaicha’s with differing degrees of senescence. (**d**) Senescence of wild-type tobacco leaves and those overexpressing pSH737-35S-*CsS40*. (**e**–**g**) Wild-type tobacco leaves and those expressing pSH737-35S-*CsS40* were treated with ethrel solution, following which the chlorophyll content, SOD-like activity, MDA content, and LOX activity were measured. WT, non-transgenic tobacco designed as a control; NtpSH, transgenic tobacco overexpressing *CsS40* (*P < 0.05; **P < 0.01).

The *S40* gene family has been shown to play a role in senescence processes in plants [25, 26]. ‘Fuding Dabaicha’ leaves of different ages (young leaves, mature leaves, and senescent leaves) (Fig. 6a) were ground into a powder and subjected to HPLC to determine the caffeine accumulation. As the senescence degree of the tea leaves increased, the caffeine content gradually decreased (Fig. 6b–c). qRT-PCR analysis demonstrated that the relative expression levels of *CsS40* in young, mature, and senescent leaves were 23.82, 49.35, and 124.13, respectively. The increasing expression level of *CsS40* significantly was clearly correlated with aging of tea leaves. We therefore speculated that CsS40 regulates caffeine content in a close relation to the senescence of tea plants.

To further verify the relationship between *CsS40* and plant senescence, fully expanded leaves of NtpSH and CK tobacco plants were plucked from the same positions of the tobacco plants and incubated *in vitro* for 14 days, the senescence of tobacco leaves was observed (Fig. 6d). Yellowing and withering appearances were only observed on NtpSH but not CK tobacco leaves, indicating that the *CsS40* overexpression transgenic plants aged faster. When the leaves of NtpSH and CK tobacco plants were treated with ethrel solution and transferred to the dark, chlorophyll content, superoxide dismutase activity (SOD-like activity), malondialdehyde content (MDA), and lipoxygenase activity (LOX activity) were measured (Fig. 6d–g). The average chlorophyll content of NtpSH was 13.73% lower than that of CK, indicating that *CsS40* overexpression accelerated the degradation of chlorophyll. The SOD activity in NtpSH plants was also lower than that of CK. Additionally, the LOX activity and MDA content in NtpSH were higher than those in CK plants, the former of which likely resulted in a decline in plant nutritional quality and the latter likely increased the degree of cell damage and accelerated senescence. In one word, *CsS40* overexpression in tobacco renders plants more susceptible to senescence.

## Discussion

### Discovery of CsS40 TF as a novel TCS1 regulator

Caffeine is of widespread interest as an economically important alkaloid in tea plants. Caffeine biosynthesis in plants is also regulated tightly by environmental and hormonal factors, which are usually mediated by various TFs via likely conserved mechanisms [15, 17]. However, so far only CsNAC7 and CsMYB184 are functionally characterized to regulate caffeine biosynthesis by up-regulating the key biosynthesis genes *NMT1* and *TCS1,* respectively [15, 17]. *CsNAC7* in tea cultivar ‘Yinghong 9’ could activate the expression of *NMT1* and increased caffeine contents [15]. CsMYB184 is highly expressed R2R3-MYB factor in tea plants, and a LTR insertion in its promoter caused low expression was responsible for low-caffeine production in a wild tea relative KeKe tea [17]. We employed *TCS1* promoter from ‘Fuding Dabai’ as the bait in Y1H assays to conduct a forward genetic screening of a yeast expression library successfully constructed from tea leaf cDNAs, and found a novel senescence-related TF, CsS40, that could bind to *TCS1* promoter. Various biochemical tests strongly supported that CsS40 bound to a short sequence of *TCS1* promoter containing several *cis*-elements. Gene expression analysis also supported that while *TCS1* expression patterns are closely related to caffeine accumulation in tea plant tissues, the expression patterns of CsS40 seemed to be opposite to *TCS1* expression and caffeine contents in tea plant tissues.

### CsS40 is a negative regulator of TCS1 expression and caffeine biosynthesis

We further conducted various molecular biological and genetic experiments to define the regulation role of CsS40 on *TCS1* gene. Because of no well-established genetic transformation technology for tea plants, we used virus-induced gene silencing (VIGS) technique, which has been established for the specific degradation of endogenous mRNAs in many plants [24], to silence the transcription of *CsS40* in tea plants. In recent years, VIGS has been successfully applied in functional genomic studies in plants. In tomato, VIGS experiments verified that silencing of *SNAC* significantly suppressed the expression of *LeACS* and *LeACO*, thereby inhibited the fruit ripening process [27]. In wheat, VIGS had been used to show that *TaRSR1* negatively regulates wheat seed starch synthesis by temporally controlling the expression of starch synthesis-related genes [28]. An effective VIGS technique was also established in tea plant leaves previously [24], and our results demonstrated that VIGS silencing of *CsS40* in tea leaves increased the transcription of *TCS1* and thereby increased the caffeine content in comparison with wild-type control leaves. Therefore, CsS40 negatively regulates caffeine content in tea plants.

Different plant organs-derived callus tissues have been widely used for genetic transformation of various genes for functional study [15]. Overexpression of *MdMYB15L* results in reduced cold tolerance in red-fleshed callus [29]. In apple, overexpression of *MdbHLH33* in transgenic tissues is able to regulate the expression of *MdCBF2* and improve cold tolerance [30]. Moreover, overexpression of *PjSTS*1a and *PjSTS2* in *Vitis amurensis* transgenic tissues increased stilbene content [31]. We further generated transgenic tea leaf calli with *CsS40*-overexpression or knockdown by genome editing. The transgenic tea leaf callus tissues overexpressing *CsNAC7* also showed an increased expression of *NMT1* and enhanced caffeine contents [15]. By contrast, we observed that overexpression of *CsS40* in tea leaf calli decreased *TCS1* expression level and reduced caffeine accumulation as compared with control tea leaf calli. Therefore, overexpression of *CsS40* resulted in a significant decrease in *TCS1* transcription level, and thereby reduced accumulation of caffeine in tea leaf calli. On the contrary, silencing of *CsS40* had the opposite effect: *TCS1* expression level increased and caffeine contents elevated in *CsS40*-silencing transgenic callus, as compared with the control. Therefore, it can be concluded that *CsS40* negatively regulated *TCS1* expression and caffeine accumulation in tea callus.

### CsS40 regulated leaf senescence, hormone signaling, and nutrient status

In plant, S40 is composed of a special gene family with diverse, but largely unknown functions, and S40 is mostly known as a leaf senescence regulating factor. As a plant-specific transcriptional regulator, S40 plays diverse roles in the regulation of phytohormone synthesis and signaling and thereby senescence process in plants. *Arabidopsis thaliana* S40–6 (AtS40–6) and *Caragana intermedia* CiS40–11 promoted the synthesis of cytokinin by inhibiting the expression of the negative regulator *MYB2* in *C. intermedia* plants [32]. In *A. thaliana* plants, AtS40 negatively regulated ABA signaling in the seed germination and early growth of seedlings [33]. However, other studies showed that S40 protein promoted leaf senescence. Rice OsS40 have been shown to play a role in carbon allocation and leaf senescence in *Oryza sativa* by modulating the expression of *SWEET* sugar transporter genes and other senescence-related genes [34]. Barley HvS40 was used as a biomarker of senescence by playing as a key upstream regulator of a well-known senescence gene *SAG* expression [35]. Although the perennial tea plants barely show any typical leaf senescence phenotypes, marked with yellowing leaves, increased ABA level, reduced photosynthesis and other physiological activities, tea leaf may undergo special leaf senescence-related physiology.

Our study also showed that *CsS40* transcription was up-regulated by JA, ABA, GA and ethylene and overexpression of *CsS40* in tobacco plants rendered tobacco leaves more susceptible to aging, such as darkness or ethylene-generator chemical treatments-induced leaf senescence, indicating that CsS40 has a promoting effect on ethylene-induced leaf senescence process. Our data further showed that *CsS40* rendered tobacco leaf more susceptible to senescence in *CsS40*-overexpression tobacco plants. Interestingly, we also detected higher xanthine and hypoxanthine levels in *CsS40*-overexpression tobacco plants than controls, indicating that CsS40 may be also involved in nitrogen metabolism, similar to what AtS40 was up-regulated in nitrogen-deficient Arabidopsis plants. We demonstrated that the caffeine content in tea leaves gradually decreased over tea leaf maturation; meanwhile, CsS40 expression level increased significantly over tea leaf maturation. These patterns are consistent with the note that CsS40 negatively regulated *TCS1* expression in tea callus and the accumulation of caffeine in tea leaves. Therefore, we speculate that CsS40 may regulate the leaf maturation of tea leaves by regulating the accumulation of caffeine.

The tobacco leaves overexpressing *CsS40* senesced more rapidly than those of wild-type tobacco plants. Our study suggested that chlorophyll contents, reactive oxygen species ROS production-triggered leaf senescence and lipid peroxidation (LOX and MDA), involving ROS scavenging genes such as *SOD*, all demonstrated that overexpression of *CsS40* in tobacco triggered aging process in tobacco leaves. It has been reported that caffeine can activate telomerase reverse transcriptase (*TERT)* gene promoter and increase *TERT* expression level, increased the telomere length and delay cell aging [36]. Interestingly, the higher *TCS1* expression level and caffeine content, but lower *CsS40* expression level in young tissues of tea plants may also be associated with delayed aging as compared with mature and older leaves with lower *TCS1* expression level and lower caffeine content, but higher *CsS40* expression level in tea plants. In the mature tea leaves with reduced the accumulation of caffeine in tea leaves may be more susceptible to aging. The opposite expression trends of *TCS1* and *CsS40* may well explain how caffeine biosynthesis slows down gradually over the tea leaf maturation process. However, these hypotheses need further investigation. Nevertheless, our study provides a new perspective for the regulation of caffeine biosynthesis in association with senescence by CsS40, and improves our understanding of the accumulation and regulation of caffeine in tea plants.

## Conclusion

Using yeast one-hybrid screening of tea leaf yeast expression library for *TCS1* promoter binding TFs, we found an unknown TF that bound to the *TCS1* promoter, which was identified as a S40 family protein in tea plants. We further characterized the functions and properties of the *CsS40* gene, including expression patterns, its coding protein binding to *TCS1* promoter and negatively regulating *TCS1* expression and caffeine accumulation in both tea leaves by using VIGS technique and in transgenic tea leaf calli overexpressing and silencing *CsS40* gene with overexpression and gene-editing strategies. In transgenic tobacco plants overexpressing *CsS40* gene, we also confirmed that CsS40 was involved in leaf senescence. This was also consistently supported by the CsS40 expression patterns in tea leaves in connection to TCS1 expression, caffeine accumulation, and leaf maturation. The study contributes to our understanding of how caffeine levels in tea leaf are controlled and what the role is of caffeine in tea plant leaf, and provides new insights into the molecular mechanism underlying the caffeine accumulation and the role related to leaf maturation and response to abiotic stress in tea plants.

## Materials and methods

### DNA extraction

The modified cetyltrimethylammonium bromide (CTAB) method [37] was used to extract total DNA. Briefly, 200 mg of different fresh tea plant leafs were ground in liquid nitrogen, lysed in ice-cold cytosolic lysis solution, and incubated at 65°C in a water bath for 15–30 min. Subsequently, 3 μL ribonuclease solution was added and incubated for a further 15–30 min at 37°C. After cooling to room temperature, 200 μL protein precipitation solution was added, and the solution was subjected to high-speed shaking for 5 min on ice. The solution was centrifuged for 4 min at 15000 × *g*, and the supernatant was transferred to a 1.5-mL enzyme-free microcentrifuge tube containing 600 μL isopropanol. After the formation of a white thread-like band of DNA, the solution was centrifuged for 1 min at 15000 × *g* and the supernatant were discarded. The DNA precipitate was washed in 600 μL 70% ethanol, centrifuged for a further 1 min at 15000 × *g*, and the supernatant was removed. After air-drying for 10–15 min, the DNA precipitate was resuspended in 100 μL DNA reconstitution solution and incubated at 65°C for 1 h. A 2-μL aliquot of ‘Fuding Dabaicha’ genomic DNA was subjected to electrophoresis on a 1% agarose gel for verification.

### Expression of *TCS1* in tobacco leaves

Tea plant *TCS1* gene sequences were screened from the Sucha tea plant genome database (http://tpia.teaplants.cn//index.html) [9] based on published *TCS1* gene sequences [11, 22]. The *TCS1* (TEA015791) gene was identified from the tea genome database (http://tpia.teaplants.cn/) and related literature. The sequence of the promoter was obtained by extending 2000 bp from the initiation codon of *TCS1* in the 5′ direction and was submitted to PlantCARE (http://bioinformatics.psb.ugent.be/) for *cis*-acting element analysis. The relative tissue expression of *TCS1* in tea plant under different hormone treatments was verified by RT-qPCR (Tables S2 and S3, see online supplementary material).

### Genetic transformation of tobacco leaves for *CsS40* overexpression

*A. tumefaciens* was transformed with the effector vectors, and an *A. tumefaciens* solution was subsequently prepared at a 1:1 ratio at an OD_600_ of 0.6 (the dilution was adjusted using 10 mmol/L AS (Acetosyringone, Solarbio, Beijing, China), 150 μmol/L MES (Ethyl methyl sulfonate, Solarbio, Beijing, China) and 10 mmol/L MgCl_2_ solution). Tobacco leaves were transformed using the leaf disk method [38]. In brief, tobacco leaves were cut into 1-cm^2^ pieces, transformed with *A. tumefaciens* solution for 8 min, and then cultured in screening medium (25°C, illumination time = 16 h/d) for 2 weeks to obtain resistant shoots of 1–2 cm in height. The bases of the oblique cuts were inserted into rooting medium to obtain resistant plants, which were cultured for 20–30 days and then transplanted to nutrient soil. The leaves of the resistant plants were cut into 0.5-cm^2^ slices, soaked in GUS staining solution at 37°C for 24 h, washed in 75% ethanol, and subjected to histochemical analysis. Untreated tobacco leaves were used as a negative control, and tobacco leaves expressing GUS were used as a positive control (Fig. S7, see online supplementary material).

### *TCS1*promoter-driven *GFP* expression in tobacco plants

The obtained resistant plants were subjected to PCR using a TaKaRa Ex Taq™ kit (Takara, Japan, China) and primers specific for GFP to amplify the gene sequence for the *TCS1* promotor (Table S3, see online supplementary material). GFP expression was detected by 1% agarose gel electrophoresis to verify successful transformation. The roots, stems, and leaves of the transgenic tobacco plants were cut into 0.5 cm-long pieces, added to embedding agent, snap-frozen for 20–30 min, and sectioned. The sections were placed in water and then sealed with 80% glycerol, after which the expression of GFP in the different tobacco tissues was observed under a laser-scanning confocal microscopy.

### Yeast expression library construction and screening of candidate transcription factors

Tea plant leaves (800 mg) were snap frozen in liquid nitrogen and then crushed. A Quick RNA Isolation Kit (Huayueyang Biotechnology, China) was used to extract total RNA. Next, a SMARTer® PCR cDNA Synthesis Kit was employed to synthesize cDNA, and an Advantage R2 PCR Kit (Clontech, San Francisco, USA) was used for amplification to yield SMART cDNA. A Chroma Spin TE400 column was used to purify SMART cDNA fragments larger than 500 bp, which were subsequently combined with the pGADT7Rec prey vector. A cDNA library was created by transforming the bait pHis2–3-AT yeast strain with the prey vector/cDNA using a ClonExpress II One Step Cloning Kit (Vazyme, Piscataway, USA). The *TCS1* promotor was amplified from the cloned plasmid using specific primers engineered to insert EcoRI and SacI sites (Table S3, see online supplementary material). The amplified products were digested with these restriction endonucleases and cloned into the pHis2 vector (Clontech, San Francisco, USA) to obtain the pHis2-Pro-*TCS1* bait plasmid. Y187 Gold yeast cells (Clontech, San Francisco, USA) were transformed with the bait plasmid and plated on synthetic dropout SD/−Trp-Leu-His media (SD-TLH). To obtain colonies resistant to 3-AT (3-amino-1, 2, 4-triazole), yeast was plated on SD-TLH media containing 0 nM, 50 nM, or 100 nM 3-AT. Transformed yeast was plated on SD-TLH media containing 50 ng/mL 3-AT, colonies were picked and DNA was extracted, which was subjected to DNA sequencing for identification. Candidate transcription factors related to the *CsTCS1* promoter were chosen for subsequent experiments. All kits were used according to the manufacturer’s instructions.

### Detection of pHis2-pro-*TCS1* self-activation

The minimum concentration of 3-AT required to inhibit the growth of yeast expressing the bait pHis2-pro-*TCS1* vector was evaluated according to the *His3* reporter gene. Well-grown monoclonal colonies (colonies >1 mm in diameter) were picked from SD/−Leu-Trp (SD-TL) plates, negative control and positive control (Fig. 1b) and plated on SD-TLH media containing a competitive inhibitor of His3 at 0 nM, 50 mM, or 100 mM. Transactivation activity was assessed by observing the growth of yeast colonies.

### Interaction between transcription factors and the *TCS1* promoter in Y1H

The candidate transcription factors were cloned into the PGADT7 vector. Y187 yeast cells were co-transformed with the pHis2-Pro-*TCS1* and PGADT7 plasmids, plated on SD-Trp/−Leu medium, and cultured for 3–4 days at 28°C. Single yeast colonies were selected and cultured in SD-Trp/−Leu liquid medium at 28°C, 200 rpm until an OD_600_ of 0.8–1.0 was reached. Yeast was subsequently cultured on SD-TLH + 3-AT (50 mM, 100 mM, 150 mM, 200 mM, or 250 mM) at 28°C for 3–4 days. Transcriptional activation of the *TCS1* promoter by candidate transcription factors was analysed by observing yeast growth.

### Split-luciferase complementation assay

pGreenII 0800-LUC vector was used to construct recombinant Pro-*TCS1*-nLUC and cLUC-35S:*CsS40* plasmids, which were used separately to transform the *Agrobacterium rhizogenes* (EHA105) bacterial strain. Transient fusion expression of recombinant plasmid in infected leaf cells via EHA105 infection was performed as previously described. After 3–5 days, the infected leaves were sprayed with 0.2 mg/mL D-fluorescein sodium salt and incubated at 37°C for 10 min. Fluorescence images were obtained using a chemiluminescence instrument Fusion FX7 (VILBER, France). The transcriptional activity based on the ratio of FLUC and RLUC was measured with a dual luciferase reporter gene assay kit.

### Electrophoretic mobility shift assay (EMSA)

cDNA of CsS40 was introduced in *p*ET32a, and recombinant His-CsS40 was purified by Ni-NTA His Bind purification Kit (Novagen, Beijing, China) according to the manufacturer’s instructions. EMSA was performed using LightshiftTM Chemiluminescent EMSA Kit (Thermo Scientific, USA). For biotin labeled-probe (Zoonbio Biotechnology, Nanjing, China) sequences (Table S4, see online supplementary material).

### Subcellular localization of the *CsS40* gene

The NCBI database was used to analyse the *CsS40* gene, and ProtParam was used to predict the molecular weight and isoelectric point of the *CsS40* protein product. Specific primers were used to amplify the full-length ORF of *CsS40* (Table S5, see online supplementary material), which was subsequently inserted into the 35S: GFP vector using the ClonExpress II One Step Cloning Kit following digestion with KpnI and XbaI (Vazyme, Piscataway, USA). *A. tumefaciens* GV3101 was transformed with the recombinant vector 35S:*CsS40*-GFP (Fig. S8, see online supplementary material) and used to infect tobacco leaves as previously described. GFP fluorescence was observed using a confocal laser-scanning microscope (Zeiss, Jena,Germany).

### Silencing of *CsS40* in ‘Fuding Dabaicha’ tea plants using VIGS

Full-length *CsS40* was cloned into the pTRV2 vector (Clontech) using T4 DNA ligase (Covent, London, UK) following digestion with *EcoR*I and *BamH*I to obtain the pTRV2-*CsS40* plasmid. *A. tumefaciens* GV3101 was transformed with pTRV2-*CsS40* or the empty pTRV1 vector (Clontech, San Francisco, USA), and an infection solution was prepared by resuspending *A. tumefaciens* to an OD_600_ of 1.2 in liquid MS medium containing 1 ml/L 6-BA, 2 ml/L AS, 0.1 ml/L NAA, and 8 g/L NaCl. ‘Fuding Dabaicha’ tissue culture seedlings were infected twice, for 5–6 min each, with an infection solution at an OD_600_ of 1.20 using the vacuum infiltration method at a pressure of 0.7 kPa. The seedlings were then transferred to a normal environment for 1 h, after which they were subjected to 0.1 ml/L 6-BA, 0.6 ml/L GA (Gibberellin A3), and 5 ml/L Chl (Chloramphenicol) hydroponic culture and incubated in the dark for 2 days [24]. It was transferred to 25°C and cultured for 35 days under light for 14 hours. CsS40 quantification and caffeine content determination experiments were performed.

### Overexpression and silencing of *CsS40* in tea callus cultures

Specific primers were used to amplify full-length *CsS40* (Table S6, see online supplementary material), which was subsequently cloned into the overexpression pSH737-35S vector and the silencing pBWA(V)K-cas9pl(atu6i)-35S vector using the ClonExpress II One Step Cloning Kit following digestion with *Xho*I and *EcoR*I (Vazyme, Piscataway, USA). The recombinant overexpression and silencing plasmids, pSH737-35S-*CsS40* (Fig. S4, see online supplementary material) and pBWA-35S-*CsS40* (Fig. S5, see online supplementary material), respectively, were used to transform *A. tumefaciens* GV3101, which prepare a *A. tumefaciens* sensitized solution.

The leaves of ‘Fuding Dabaicha’ tissue culture seedlings were cut into small pieces of approximately 1 cm^2^, treated with 100 μmoL/L AS (Acetosyringone) for 20 min, and transferred to the *A. tumefaciens* sensitized solution for a further 10 min. The leaves were dried by blotting with sterile filter paper and then incubated on solid MS medium containing 0.5 mg/L 6-BA (N-(Phenylmethyl)-9H-purin-6-amine) and 1.0 mg/L NAA (1-naphthlcetic acid) for 3 days in the dark. Subsequently, the leaves were transferred to solid MS medium containing 0.5 mg/L 6-BA, 1.0 mg/L NAA, 200 mg/L Tim, and 30 mg/L Kan, which was changed every two weeks. After approximately two months, the leaves were subjected to PCR and DNA sequencing analysis.

### Determination of caffeine, xanthine, and hypoxanthine in plant leaves

To determine the amount of caffeine, xanthine, and hypoxanthine in each sample, we first added 5 mL of a 70% methanol solution to 0.2000 g dried leaf powder at 70°C for 10 min and repeated this extraction once. After centrifugation (7104g, 4°C, 10 min), the supernatants from the three extractions were collected and combined. After 0.45 μm filtration, the tea infusion underwent HPLC analysis using a TC-C18 column (250 mm × 4.6 mm, 5.0 μm) (Agilent Technologies, Santa Clara, CA, USA).

Caffeine: The injection volume was 10 μL, flow rate 1 mL/min, and temperature 35°C. The mobile phases were (i) 0.05% acetic acid aqueous solution, (ii) 100% acetonitrile, and (ii) 100% methanol, and the gradient elution started at 85%A/5%B/10%C (v/v) and was maintained for 5 min, then changed to 70%A/10% B/20%C (v/v) for 10 min, and then maintained at 72%A/8% B/20%C for 25 min for re-equilibration, ultraviolet detector at 278 nm.

Xanthine and hypoxanthine: The injection volume was 10 μL, flow rate 1.2 mL/min, and temperature 35°C. The mobile phases were (i) methanol, (ii) 0.01 mol/L ammonium dihydrogen phosphate, and the gradient elution started at 7%A/93%B (v/v) and was maintained for 30 min, ultraviolet detector at 254 nm.

### RT-qPCR verification of transgenic tea callus tissues

DNA extraction from tea plant tissues was performed as described above for tea plant leaves. A Plant RNA Extraction Kit (Quick RNA isolation Kit; Haidian District, Beijing) was used to extract total RNA. qRT-PCR was conducted to evaluate the expression patterns of *TCS1* and *CsS40* using SYBR Premix Ex Tag (Takara, Japan, China). The −ΔΔ^CT^ method was used for analysis and expression levels were normalized to *GAPDH*. All experiments were performed three times in triplicate.

## Supplementary Material

Web_Material_uhad162
