# Supplementary material for: The transcription factor CsS40 negatively regulates TCS1 expression and caffeine biosynthesis in connection to leaf senescence in Camellia sinensis
Source: Hortic Res. 2023 Aug 10;10(9):uhad162. doi: 10.1093/hr/uhad162 (PMC10508035; doi:10.1093/hr/uhad162)
Supplement: Web_Material_uhad162 [file web_material_uhad162.zip › Supporting Figures.docx]

The transcription factor CsS40 negatively regulates *TCS1* expression and caffeine biosynthesis in connection to leaf senescence in *Camellia sinensis*

Xinzhuan Yao ^1^, Hufang Chen ^1^, Antao Ai ^1^, Fen Wang ^2^, Shanshan Lian ^1^, Hu Tang ^1^, Yihe Jiang ^1^, Yujie Jiao ^1^, Yumei He ^1^, Tong Li^1^, Litang Lu ^1*^

1 College of Tea Sciences, Institute of Plant Health & Medicine, The Key Laboratory of Plant Resources Conservation and Germplasm Innovation in Mountainous Region (Ministry of Education), Guizhou University, Guiyang 550025, China

2 School of Biological Science and Agriculture, Qiannan Normal University for Nationalities, Duyun, China

*Correspondence: [ltlv@gzu.edu.cn](mailto:ltlv@gzu.edu.cn) ; Tel.: +86-13639031105

*Corresponding author at: College of Tea Science, Guizhou University, Guiyang 550025, People’s Republic of China.

E-mail addresses: [xzyao@gzu.edu.cn](mailto:xzyao@gzu.edu.cn) (X. Yao), [15761629978@163.com](mailto:15761629978@163.com) (H. Chen), [1248186751@qq.com](mailto:1248186751@qq.com) (A. Ai), fenmin521@163.com(F. Wang), [2453102127@qq.com](mailto:2453102127@qq.com) (S. Lian), [htang@gzu.edu.cn](mailto:htang@gzu.edu.cn) (H. Tang), [yhjiang@gzu.edu.cn](mailto:yhjiang@gzu.edu.cn) (Y. Jiang), [yjjiao@gzu.edu.cn](mailto:yjjiao@gzu.edu.cn) (Y. Jiao), [ymhe@gzu.edu.cn](mailto:ymhe@gzu.edu.cn) (Y. He), lit@gzu.edu.cn (T. Li), [ltlv@gzu.edu.cn](mailto:ltlv@gzu.edu.cn) (L. Lu *).

>TCS1( TEA015791) promoter sequence

CCTGCTCGCCCTAGCATGATTATTAATATATATTATGTACATGTAAAATTTTACTATAAATACTTCTTATTGTATGTATTCTTAACTTATATTATACTTTTAAGTTATACAAATTTTTTTTTTCAGTTATAATTAATTTTTTTTTCTGTAGTTGCGATCTTATTTTAGTTATGACTCATAATATCTCATGTATTATTTGGTTGACTTTTTTTTTTTTTTTGACTTTAAAATATGAAATTATGTCATTTTTTTTTTAGTTATAAGGTGCATACGATACATACCCTTTAAAATTTTCTTGTATGTACATATGTACATATTAATATCTGTATATATATAATTTACAAAATTTTACTTTTTTAGAATAAAAAGATATACTTAAAATGAAAAATGTATTTATTTAAGTTAATATTTAAGATGTAATGACTAAATATTAAGGTGTAATTTTAATTAGCTCGATTTTGATTTTAAATTTTTAACTTTGTCTCAATTTGGTCAAATGGATTACAACTCTTAATATTTTTTAATAAAAAAATATGTATATAAGTGTCATGTGTCTCAAAATTATGAATTTATCTAACTCAGTAAGTAAACCTCAACTAATTTAGTGATAATTTTTCTTAAAAAACACTAATGATATTATTAATTTGATCAAATTGACGTAAAATCTAAAATTAAGGATCAAAATCAAATCAATTAAAAATGTAAAAACTAAAATAAAACAAAAGATAAAATATAAGGACATCCGTGTAATTCACCCACAAAATTATCATTTTTCAGTTTTATAATATTTTAAATTGTTTATATGAGTTTGTTGGGCAAGTTCGAGATTGTACTAGCAAGATTTTAACGCTAGCTTGGGAGGGATTTTGTGTTTGTTTGATTTGTATCTCATGATATAATTTTTAATTTTTATTTTTTTAAATTTTGTTTGGTTTAAATATTTTGAATTTTTTTTTTCAAAATCACTTTTTTTTCTGTATATATCAATCAGTCACTTTTTCTTTCTTATCTTTTCTTCTCAATCATTTTTTTTTCTCACACACATCTACTCAAACTACAATAAAATATCAAATCATCCCAAAATCTCAAATTATTTTCAAAATATACAACCAAACAAACTAAAAAAATTTCTAAACTATCTCTCAAAAAAAAAAAAAAATTAAAAATTTATCTCAAAACAAAAACCAAACACACCCTTTTAGTTTCAAAAACTGAAAAAATATTTGGTTTTATTGGACGTCACGTGGCGTACTACTTACCAATAATAATATGTCATGTTTCTATTATTTTTTAACCACTTAATATAAAATTATAAATCTCATTTTTTTTATTAATTAAAATACTTGTGTATCACGTGCAAAATCAACCAATAATATCTCCAAAAAAAAATCCTAACTTAGGCGTACCCGAGCACCCAGACTATAGAGAGGCCTTCAGGCCATTATTCACATCACTGCTGTGGTAGCTGGCCTCTTTGCTATAAAAATTAGTGCTTTTCTGGTTATTCATATTCATATCACTGCTGTGGCAGCTGGCCTCTTTGCTATAAAAATTACTTTTCTGACGAGGC

> TCS1( TEA015791)

ATGGCGCTAGCTACTACGGGGAAGGTGAACGAAGTGTTGTTCATGAACAGAGGAGAAGGAGAAAGTAGTTATGCACAAAACTCTTCTTTCACGCATCAAGTGGCCTCAATGGCACAGCCAGCGCTAGAAAATGCAGTTGAAACTCTCTTCTCCAAAGATTTCCACCTTCAAGCTCTTAACGCAGCGGACTTGGGTTGTGCAGCGGGTCCAAACACATTCGCAGTGATTTCTACGATCAAGAGAATGATGGAAAAGAAATGCAGGGAATTGAATTGCCAAACACTGGAACTTCAGGTTTACTTGAATGATCTTTTTGGAAATGATTTCAATACCCTCTTCAAAGGCCTGTCGTCTGAGGTTATTGGTAACAAATGTGAGGAAGTTTCGTGTTATGTGATGGGAGTACCGGGGTCTTTCCATGGCCGGCTTTTTCCTCGTAACAGCTTACATTTAGTTCATTCCTCTTACAGTGTTCATTGGCTTACTCAGGCACCAAAAGGACTCACAAGCAGAGAAGGCTTGGCATTAAACAAGGGGAAGATTTACATATCAAAGACAAGCCCTCCTGTTGTAAGAGAAGCCTACTTATCTCAATTTCATGAAGATTTCACAATGTTTCTCAATGCTAGATCC

CAAGAGGTGGTTCCAAATGGTTGTATGGTGTTGATACTTCGTGGTAGGCAATGTTCTGATCCTTCAGACATGCAGAGCTGCTTTACTTGGGAACTATTAGCTATGGCCATTGCTGAATTGGTTTCACAGGGATTGATAGATGAAGATAAATTAGACACCTTCAATATACCCAGCTATTTTGCATCACTTGAGGAAGTGAAAGATATAGTGGAGAGGGACGGATCATTCACAATTGATCATATAGAGGGGTTTGATCTTGATAGCCTAGAAATGCAGGAGAATGATAAATGGGTTAGAGGGGAAAAGTTTACCAAGGTTGTCAGGGCCTTCACAGAGCCTATAATTTCAAACCAGTTTGGACATGAAATCATGGACAAACTATATGACAAATTCACTCACATTGTAGTTTCAGATTTGGAAGCAAAGCTACCGAAGACCACAAGTATCATCCTAGTGCTTTCCAAGATTGATGGATAG

Amino Acid Sequence：

MALATTGKVNEVLFMNRGEGESSYAQNSSFTHQVASMAQPALENAVETLFSKDFHLQALNAADLGCAAGPNTFAVISTIKRMMEKKCRELNCQTLELQVYLNDLFGNDFNTLFKGLSSEVIGNKCEEVSCYVMGVPGSFHGRLFPRNSLHLVHSSYSVHWLTQAPKGLTSREGLALNKGKIYISKTSPPVVREAYLSQFHEDFTMFLNARSQEVVPNGCMVLILRGRQCSDPSDMQSCFTWELLAMAIAELVSQGLIDEDKLDTFNIPSYFASLEEVKDIVERDGSFTIDHIEGFDLDSLEMQENDKWVRGEKFTKVVRAFTEPIISNQFGHEIMDKLYDKFTHIVVSDLEAKLPKTTSIILVLSKIDG

> *CsS40*

CGGCAACAAGCAATACCATACGACGTACCAGATTACGCTCATATGACAAGTTTGTACAAAAAAGTTGGCTAATGGAAGACCTATAGATATATATAGAATCTGTGCATATTCTTATAATTTAGATACAGAGTCCTGAAAAATCAGGACTCCGGTCGCTAGCTACGGTGGTTTTAGTGGTGGTGGTGGTGGTGATATATGGCGAAGGGTCGGAAACTAACAACTAGTCGCAGCGATCGTCTGTTGGGCCGATACAACTACGGTCAGGCCCAAGGAATGGTGACCGAGTCATCGGAGCTCGGTGAAGAGGACGTGTGGTCGATGGTCGATGACATGGTCAACGGCGGCGAAAATCACTCAATGGGCAGTTCCGGAGGCGCGTGGAGCCCACGCGCCGCCGCGGAGAGTAACGGGTCCATGAACTACCTAAACCGCCGGCACGTCGTTCCCCGTGAGGAGAGCCAACATGTGGGCGGGCTGTCACTGGCCTTTGAGGATTCCGGAAAAACGGCGTCGTCTAGGATCCTGCACCAATTCCGTACCCAGGACAGCATGGCGAGCCCACATGGTGGACGACATATGGCCACGTCAGCCCCAGTGAACGTGCCTGATTGGTCAAAGATTTACCGAGTTGACTCGGTCGAGTCGCTGCATGACTCGGATGACGGCGTGGAAGATCGGGACTCGGAGATGGTTCCGCCACACGAGTTCTTGGCGCGTGAGTACCGGAGGATGGCGGCGAAGTCGGTTTTCGAAGGTGTGGGCCGGACGCTAAAAGGCCGGGACTTGAGCCGGGTTCGGGATGCCGTGTGGAGCCAAACCGGATTTAATGGCTAAAACCATTATTGTTATTAATGCATCTTTTAAGAAGTGATTTAAAGGTTAGTTGGGTTGGTGGATTTTATTGCTAAATTGTTTTGGGGTGGGCAATGACTCGACCGAGTGAACTCAGAGAGAACGAATCAACTCGGCAACGACTGAGCCCAACGAGAGGTGTCTCCGTCTCTCATCTACTCTGTCTTTAGTAGTTTTTGGTGGTGTCTGAGCTGGCATTTCGAAAATTGTGATGAGTCTGTCAGTGGTAAGTCGGGTCATTCCAGTACTATATGTTGCTCGACCATAGAATGATAGTGTGCAGGGCATGCAATTTTGGAGCCTAGG


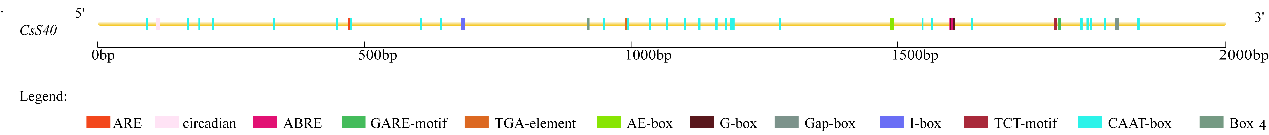


**Supporting Figure S****1 *CsS40* gene promoter sequence Cis-acting element analysis.**


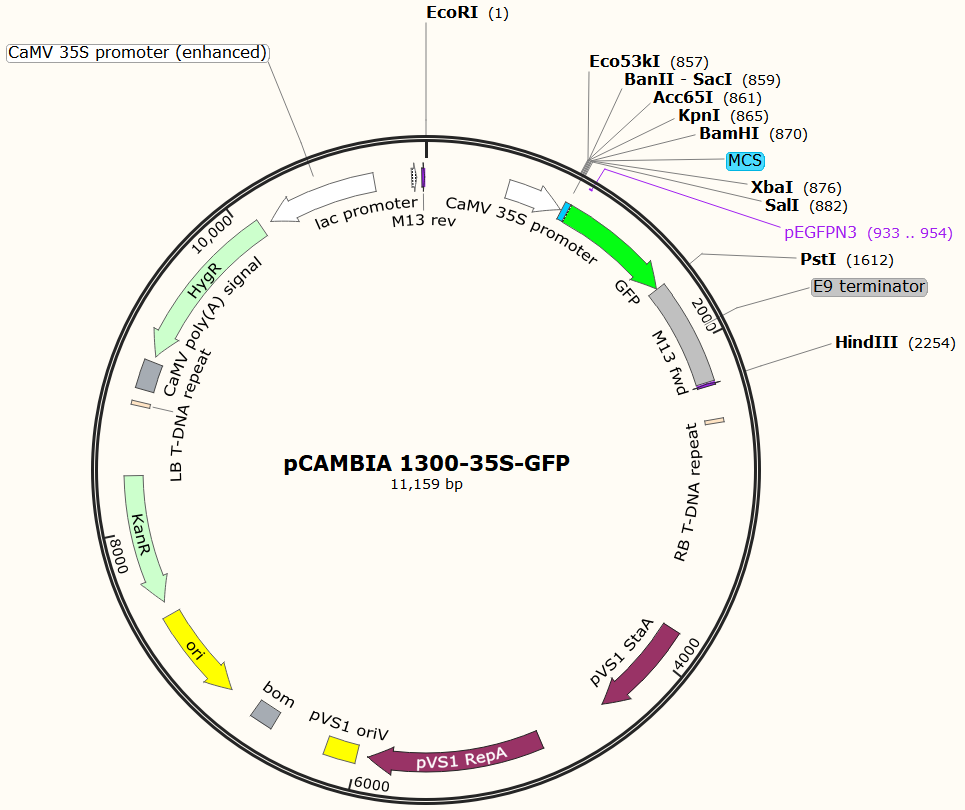


**Supporting Figure S2** ***CsS40* gene GFP vector construction.**


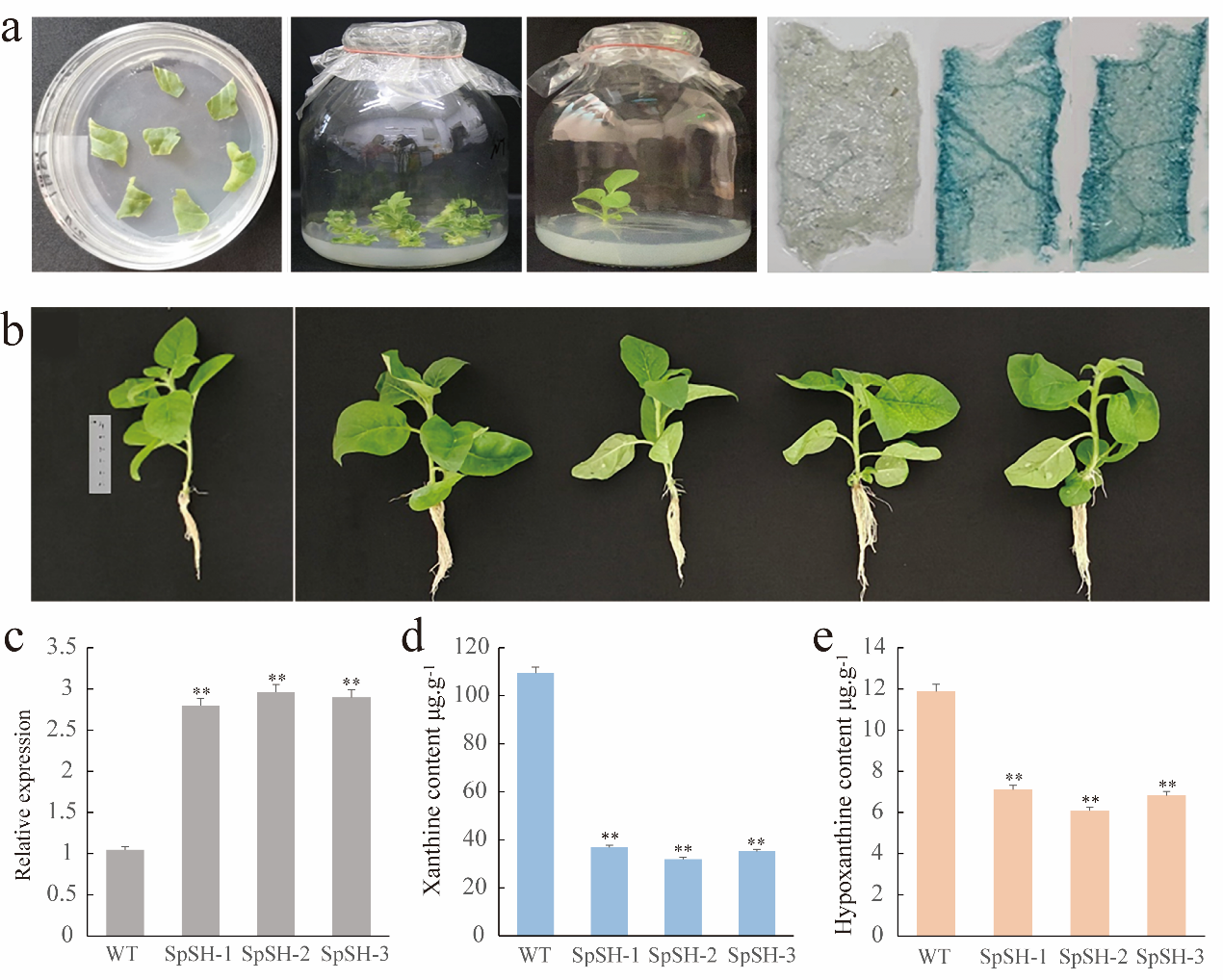


**Supporting Figure S3** **Overexpression of *CsS40* in tobacco.**

(a) Growth of tobacco leaves overexpressing pSH737-35S-*CsS40* in medium. Finally, GUS staining was performed in callus of transgenic tobacco. (b) The first tobacco seedling was wild-type, and two to five tobacco plants overexpressing pSH737-35S-*CsS40*.

**a b**


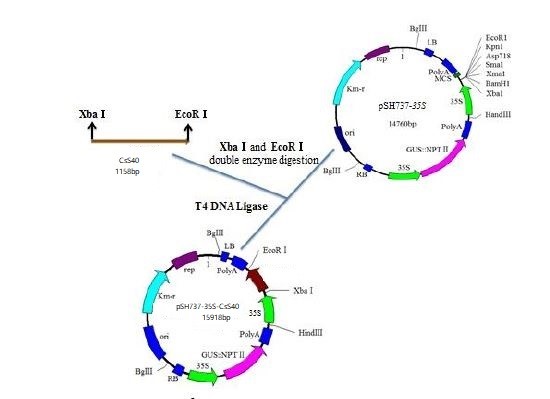

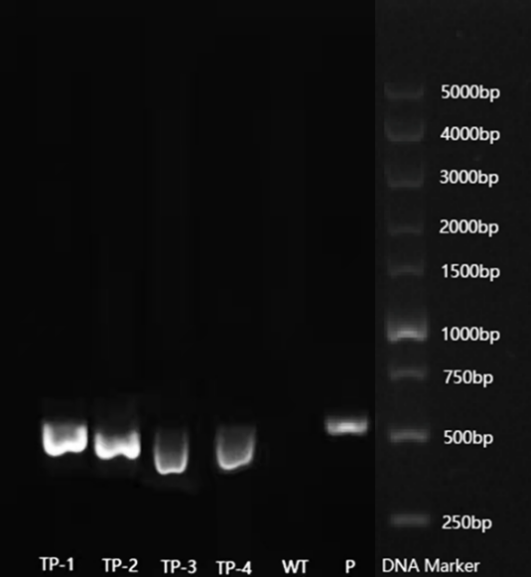


**Supporting Figure S****4 Overexpression *CsS40* in** **tea calli.**

(a) Overexpression vector map of *CsS40.* (b) PCR identification of overexpressing tea plant callus; Note: TP1-4: Transgenic tea calli 1-4; WT: wild type tea calli plants; P: Plasmid positive control


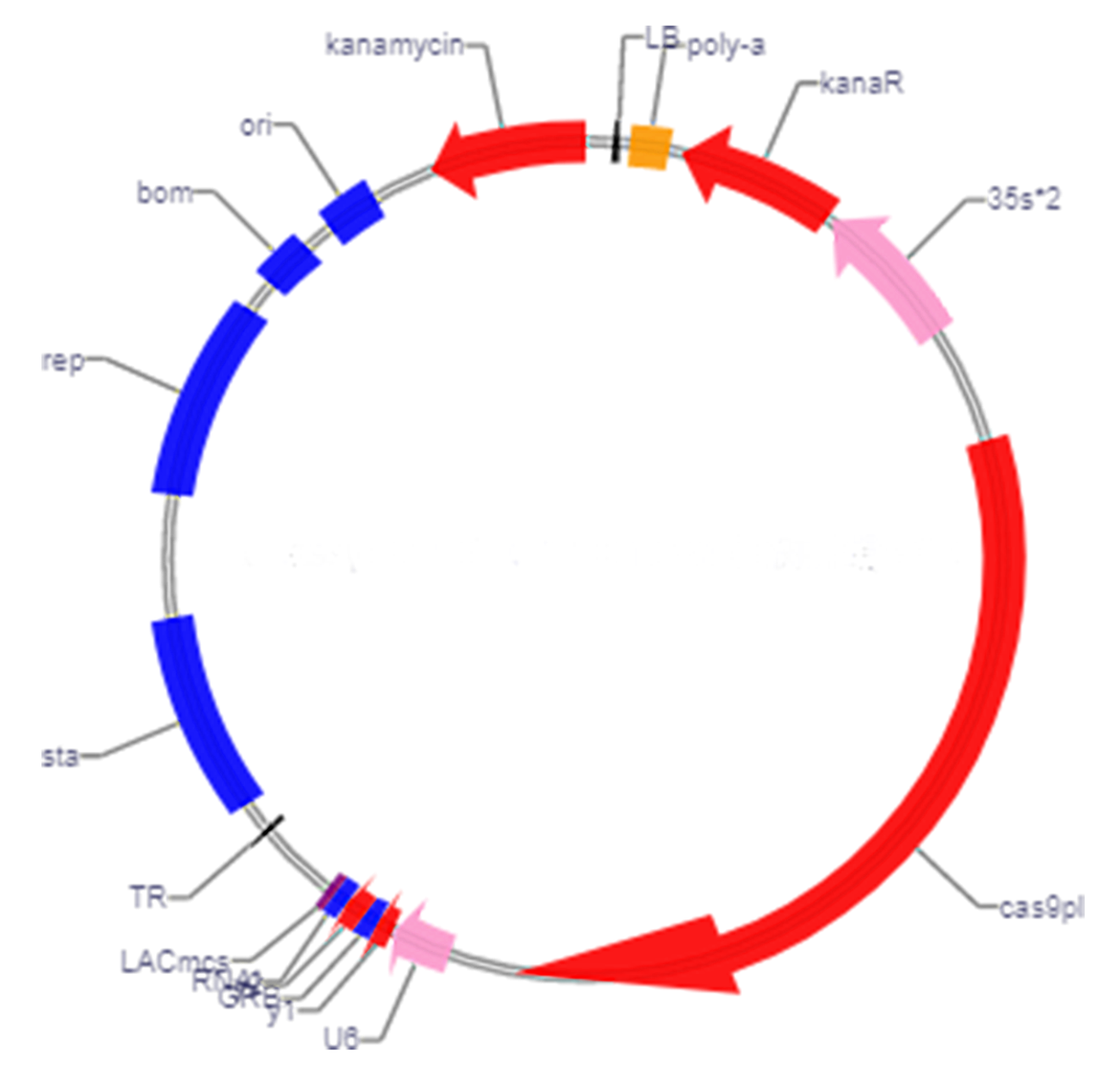


**Supporting Figure S5** ***CsS40* gene editing vector map.**


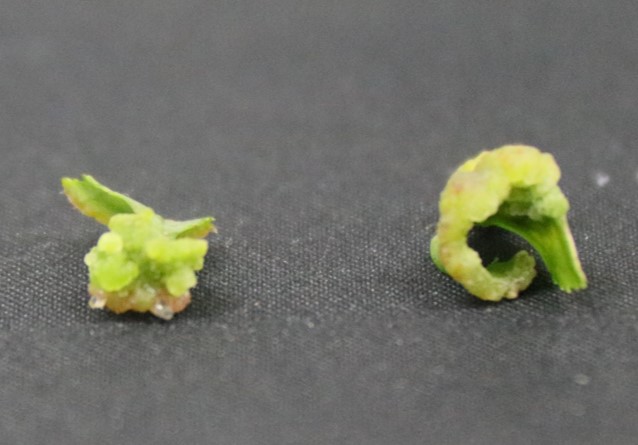

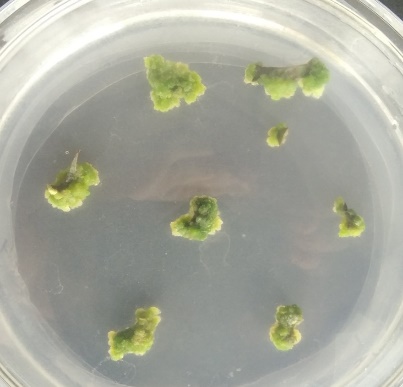

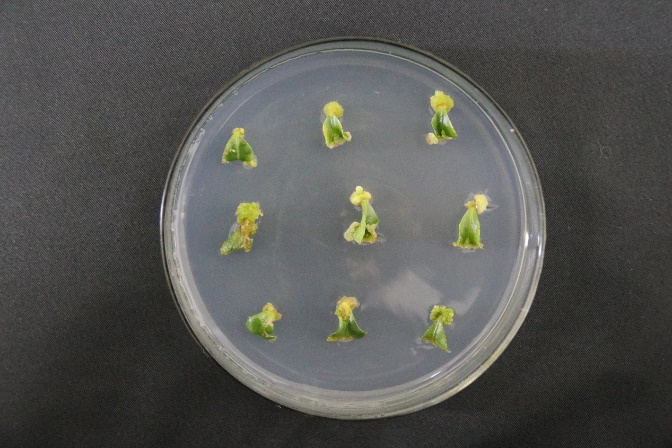
a

b


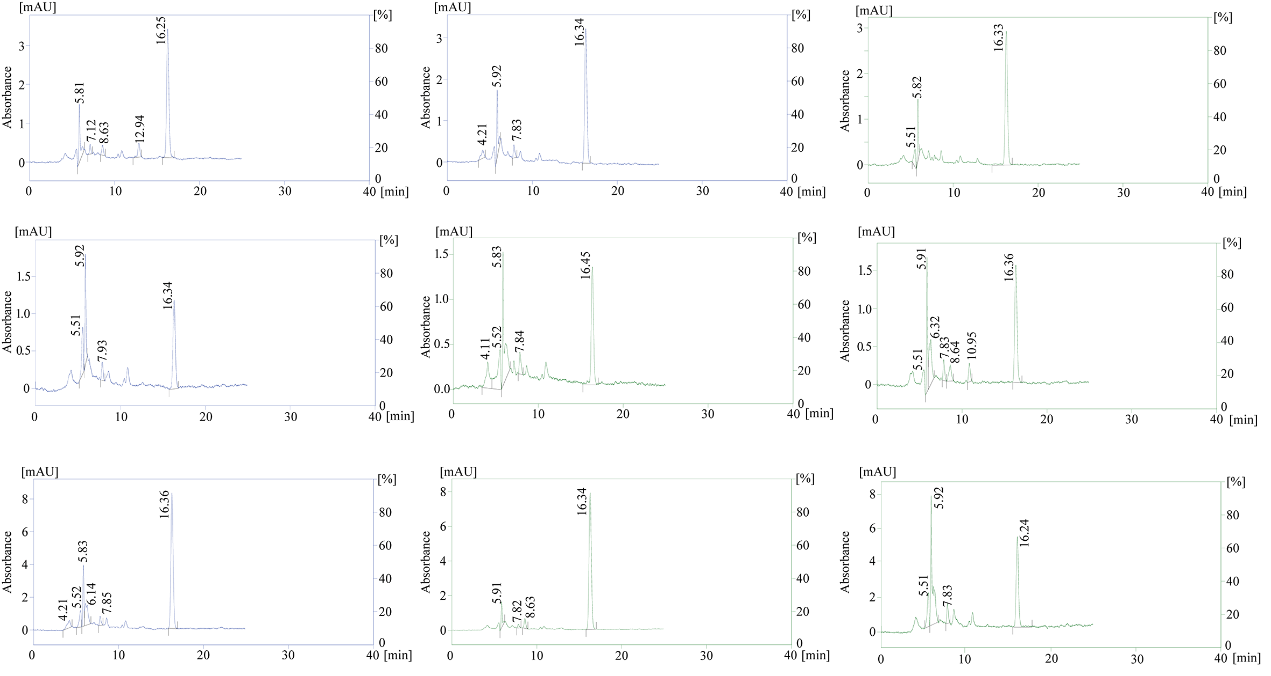


**Supporting Figure S6** **Overexpression and silencing of *CsS40* in tea callus**

(a) Tea leaf callus formation process pictures*.* (b) HPLC of caffeine accumulation in tea callus.


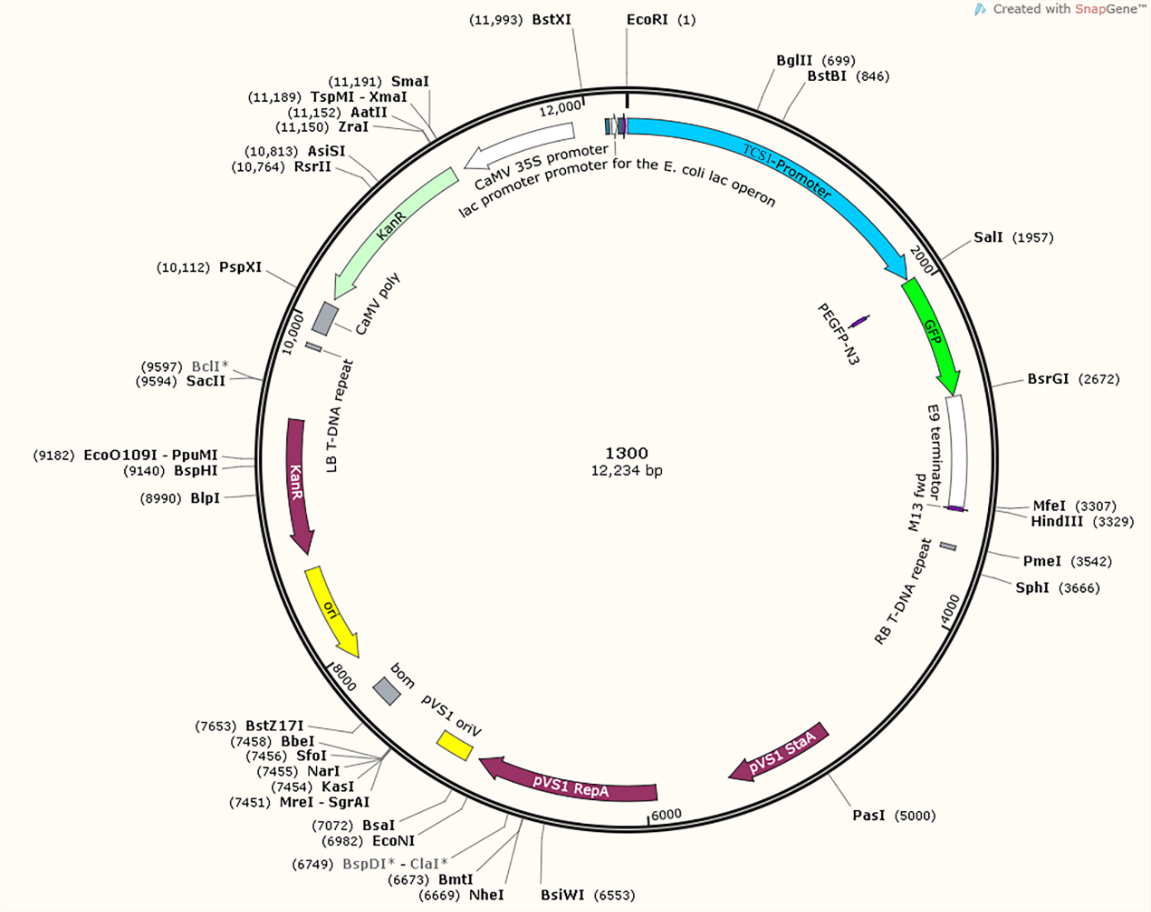


**Supporting Figure S7** ***TCS1* gene GFP vector construction.**


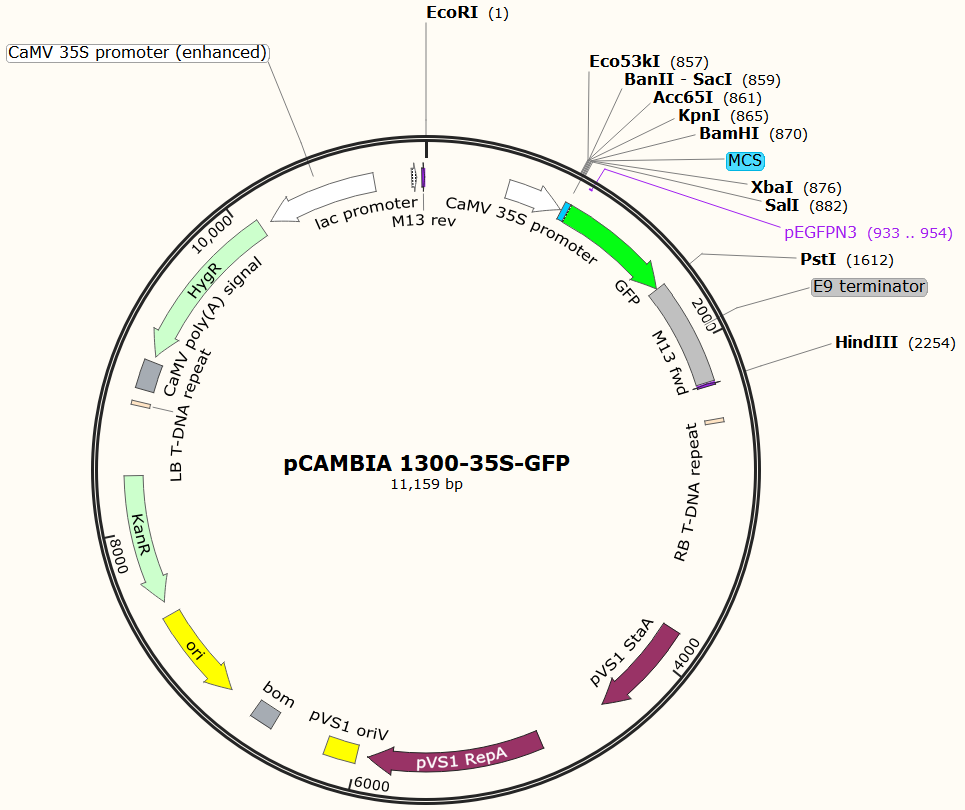


**Supporting Figure S****8 Subcellular localization vector map of *CsS40.***
